# Reduced Attentional Scope in Cocaine Polydrug Users

**DOI:** 10.1371/journal.pone.0006043

**Published:** 2009-06-25

**Authors:** Lorenza S. Colzato, Wery P. M. van den Wildenberg, Bernhard Hommel

**Affiliations:** 1 Institute for Psychological Research & Leiden Institute for Brain and Cognition, Leiden University, Leiden, The Netherlands; 2 Amsterdam Center for the Study of Adaptive Control in Brain and Behaviour (ACACia) Psychology Department, University of Amsterdam, Amsterdam, The Netherlands; University of Granada, Spain

## Abstract

Cocaine is Europe's second preferred recreational drug after cannabis but very little is known about possible cognitive impairments in the upcoming type of recreational cocaine user (monthly consumption). We asked whether recreational use of cocaine impacts early attentional selection processes. Cocaine-free polydrug controls (n = 18) and cocaine polydrug users (n = 18) were matched on sex, age, alcohol consumption, and IQ (using the Raven's progressive matrices), and were tested by using the Global-Local task to measure the scope of attention. Cocaine polydrug users attended significantly more to local aspects of attended events, which fits with the idea that a reduced scope of attention may be associated with the perpetuation of the use of the drug.

## Introduction

Taking cocaine by snorting route is Europe's second preferred recreational drug habit after smoking cannabis [1]. Given the addictive properties of this psychostimulant drug, the recreational use of cocaine is a public health issue in Europe as it is in the USA [2]. It is well known that in the long term, chronic (i.e., daily) use of cocaine is associated with reduced functioning of Dopamine D2 (DAD2) receptors [3] and dysfunctions in the serotonergic and the glutaminergic system [4], [5], in lateral prefrontal cortex (LPFC), orbito-frontal cortex [6], [7], anterior cingulate, and the cerebellum [8]. Given the key role of the frontal lobe in cognitive control [9], it is thus not surprising that chronic cocaine users, compared to non-users, show a poorer ability to inhibit their overt responses [10], perform worse on tasks measuring mental flexibility [11], [12] and conflict-control [13], and show compromised ability to control their attention [14].

Only in the recent two years, some studies have systematically looked into cognitive impairments among recreational cocaine users who do not meet the criteria for abuse or dependence but take cocaine (preferably by snorting route) on a monthly frequency (1 to 4 gram). Colzato, van den Wildenberg, and Hommel [15] found that the spontaneous eyeblink rate, a marker of dopaminergic functioning [16], [but see 17], is significantly lower in recreational users than in cocaine-free controls, suggesting that even the recreational use of cocaine is associated with hypoactivity in the subcortical dopamine system. Consistent with this picture, Colzato, van den Wildenberg, and Hommel [18] observed in a stop-signal task [19] that response inhibition, but not response execution, is impaired in recreational cocaine users. Moreover, Colzato and Hommel [20] found that, relative to a sample of cocaine-free controls, recreational users show normal sensorimotor integration, but no reliable inhibition of return [21]- the (commonly robust) phenomenon of slowed responding when attention needs to return to a previously attended location [22]. While recreational cocaine users performed significantly worse than cocaine-free controls on tasks tapping cognitive flexibility, they however show comparable performance in the active maintenance and monitoring of information in working memory (WM) [23].

It is important to consider that the causal relation between cocaine use and cognitive control functions is not necessarily straightforward or linear, as pre-existent neuro-developmental factors cannot be excluded. Recent evidence showed, for instance, that monkeys having pre-existing lowered D2 receptor densities run a higher risk to use cocaine and to become addicted [24] and that chronic users may suffer pre-existing problems in inhibitory control [25] and impulsivity [26]. However, it should be noted that the connection between cocaine, DAD2 pathways, and difficulties in inhibitory control seems robust.

Whereas previous studies on recreational use of cocaine have focused on inhibitory control, “shifting” between tasks and mental sets, and the active maintenance and monitoring of information in WM, in the present study we investigated whether even earlier attentional selection processes may be affected. Considering that cocaine use is associated with impairments in the functioning of dopamine receptors, there are a number of reasons suggesting that cocaine might impact early aspects of attentional functioning. Animal models and patients studies including Parkinson's and Huntington's disease, schizophrenia and attention deficit hyperactivity disorder (ADHD), pathologies associated with abnormal dopaminergic levels, suggest that disturbances in attentional process (typical for those pathologies) may be modulated by dopamine (see [27] for a review).

To measure attentional selection processes, we used an adapted version of the Global-Local task developed by Navon [28], which indexes how fast people can process global and local characteristics of hierarchically constructed visual stimuli (e.g., larger letters made of smaller letters). Typically, this task gives rise to the “global precedence” effect, which means that global features can be processed faster than local features. Global precedence is supposed to reflect a bias towards a large “scope” of attention, so that a small global precedence effect would imply a reduced attentional scope. There are a number of reasons why we speculated that consuming cocaine and being exposed to it may lead, among other things, to a bias towards decreased attentional spotlight. Cocaine use is often associated with compulsive drug-seeking and drug-taking behaviors. Interestingly, it has been suggested that compulsive behavior is linked with a cognitive style focused on small details in the surroundings [29]. Moreover, it has been shown that mood affects the breadth of the attentional scope, with more positive mood leading to the processing of an increased number of peripheral stimuli [30]. Given that positive mood is assumed to temporarily increase the dopamine level [31], this implies a positive correlation between dopamine level and attentional scope. Considering that cocaine use is associated with impairments of dopamine receptors, it makes sense to assume that the attentional scope may be reduced in users. Following this reasoning, we hypothesized that cocaine polydrug users as compared to cocaine-free polydrug controls might show a less pronounced, if any, global precedence effect. Given the link between mood and dopamine, we used an affect grid [32] to check whether our results might be confounded by mood differences between the two groups.

## Results

The two groups did not differ in mood, as indicated by the affect grid's valence measure (Cocaine Polydrug Users: M = 5.6, Cocaine-free Polydrug Controls: M = 5.8), F(1, 34)<1, and arousal measure (Cocaine Polydrug Users: M = 5.9, Cocaine-free Polydrug Controls: M = 6.1), F(1, 34)<1.

**Figure 1:**
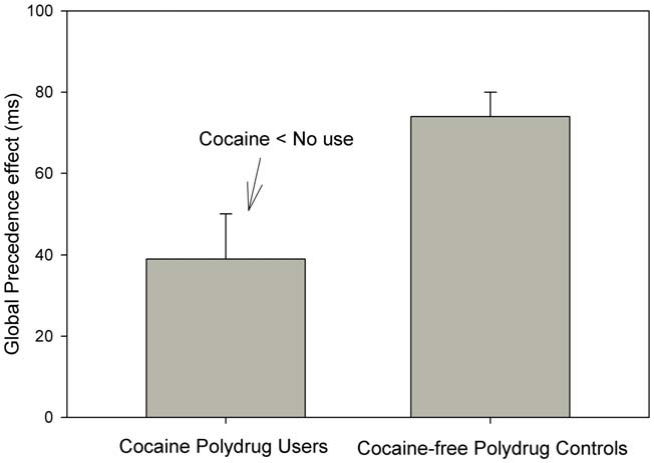
Mean Global Precedence effect for Cocaine Polydrug Users and Cocaine-free Polydrug Controls. Vertical capped lines atop bars indicate standard error of the mean.

The square roots of error percentages and median reaction times were analyzed by means of analysis of variance (ANOVA) using Target Level (global vs. local) as within- and Group (Cocaine Polydrug Users vs. Cocaine-free Polydrug Controls) as between-participants factor. The reaction time analysis showed a main effect of Target Level, F(1,34) = 79.73, p<.001, MSE = 2857.354, η2p = 0.71, which was modified by Group, F(1,34) = 7.85, p = .008, MSE = 2857.354, η2p = .19. The main effect indicated global precedence [28]: Global targets were responded to faster than local targets. However, as expected, the size of this effect varied with Group: Cocaine Polydrug Users showed a smaller, but still significant, F(1,17) = 12.26, p = .003, MSE = 4376.883, η2p = .42, global precedence effect than Cocaine-free Polydrug Controls (see Figure 1 and Table 1). Error percentages did not reveal any reliable effect, Fs(1,34)<1.60, ps>.21.

**Table 1:** Demographic characteristics, use of other recreational drugs and performance on globally and locally defined targets (SEM between parentheses).

	Cocaine-free polydrug controls	Cocaine polydrug users
N (M∶F)	18 (17∶1)	18 (15∶3)
Age (years)	25.4 (2.9)	24.3 (4.2)
Raven IQ	110.0 (4.2)	110.2 (4.4)
Monthly drinks	58.7 (28.8)	79.5 (56.1)
Monthly cigarettes**	95.6 (186.7)	202.8 (220.7)
Monthly exposure cannabis**	4.5 (3.5)	17.5 (26.5)
Lifetime exposure MDMA**	5.3 (4.5)	68 (114)
*Global Targets*		
Reaction Times (ms)	394 (25)	447 (25)
Error Rates (%)	5.1 (2.4)	9.1 (2.4)
*Local Targets*		
Reaction Times (ms)	468 (19)	486 (19)
Error Rates (%)	5.6 (1.6)	5.5 (1.6)
Global Precedence Effect **	74 (6)	39 (11)

Raven IQ, IQ measured by means of the Raven Progressive Matrices; Monthly drinks, monthly number of standard alcoholic drinks; Monthly cigarettes, monthly standard cigarettes smoked; Monthly exposure cannabis, monthly consumption of cannabis; Lifetime exposure MDMA, lifetime consumption of MDMA (ecstasy) tablets.

** *p*<0.01.

We further tested whether the use of MDMA, cannabis, alcohol, and cigarettes contributed to the effect on the global precedence. An ANOVA with group as independent variable and use of MDMA, cannabis, alcohol, and cigarettes as covariates indicated no such contribution: the effects of the covariates were far form significant, for all, F<1, and the Group X Target Level effect remained reliable, F(1,32) = 4.62, p = .039, MSE = 2821.94, η2p = 0.13. To rule out possible interactions between covariates we also ran separate ANOVAs with only one covariate each, but the covariates were still insignificant, F<1, and the target level-by-group interactions were still reliable: F(1,33) = 4.88, p = .040, MSE = 2725.98, η2p = 0.12; F(1,33) = 4.55, p = .044, MSE = 2631.96, η2p = 0.12; F(1,33) = 4.38, p = .046, MSE = 2425.55, η2p = 0.12; F(1,33) = 4.95, p = .039, MSE = 2931.34, η2p = 0.12; for MDMA, cannabis, alcohol, and cigarettes, respectively.

## Discussion

This study tested, for the first time, whether the recreational use of cocaine is associated with a detectable impact on attentional process. Cocaine polydrug users showed, compared to cocaine-free polydrug controls, a smaller global precedence effect indicating a reduced scope of visual attention. Our results fit with previous studies on chronic cocaine users which reported compromised ability to control their attention [14].

As our participants were screened for several psychiatric disorders, we can rule out an account in terms of pre-existing psychiatric disorders (such as schizophrenia, ADHD, and obsessive compulsive disorder) that have been associated with dopaminergic abnormalities [33]–[35]). Particularly important was the matching of the age range and of mood. Developmental studies indicated that the global precedence effect is unrelated to general intelligence but changes with age [36]) and it has been shown that mood affects the breadth of the attentional scope, with more positive mood leading to the processing of an increased number of peripheral stimuli [30]. Given that group differences in terms of scope of attention remained significant after MDMA and cannabis had been used as covariates, we doubt that our results can be attributed to the use of these other drugs. Indeed, even though the difference between groups in MDMA lifetime exposure and cannabis monthly consumption was very large, it is well known that MDMA and cannabis affect WM and flexibility, respectively [7], [37], [38], but not attentional processes.

Even though participants' compliance with the instruction not to take psychoactive drugs for at least two weeks was encouraged by taking a (not further analyzed) saliva sample at the beginning of the session, a reliable deceptive method often used in psychopharmacology studies [39]–[41], we cannot rule out possible acute cannabis and cocaine effects that may have confounded our results. Our findings also raise the question whether this specific attentional deficit is a risk-factor for cocaine use, or whether it predicts relapse to cocaine use among cocaine users seeking treatment.

In any case, the seemingly small amount of cocaine involved in the present findings, together with previous results showing that the recreational use of cocaine is associated with impairments in inhibitory control and flexibility [18], [21], are worrying. Everyday behavior arguably requires an “open” scope of attention in order to be able to adapt and to restructure in response to changing environmental demands [42], so that a lack of such an open scope is likely to hamper the adaptivity of recreational users on a daily basis.

Moreover, our findings have important implications for the treatment of cocaine use. A reduced scope of attention may be associated with the perpetuation of the use of the drug, and may help explaining why it is so difficult for cocaine users to change their compulsive drug-related habits and to enter and stay in rehabilitation therapy.

## Materials and Methods

### Participants

Thirty-six young healthy adults (32 man and 4 women) served as participants for financial reward and constituted the two groups: cocaine polydrug users and cocaine-free polydrug controls. The sample, that did not participate in previous studies by the authors, was drawn from adults in the Leiden and Rotterdam metropolitan area, who volunteered to participate in studies of behavioral pharmacology. Participants were recruited via ads posted on community bulletin boards and by word of mouth. Following previous work [18], [20] we made sure that the users met the following criteria: (1) a monthly consumption (1 to 4 gram) by snorting route for a minimum of one year; (2) no Axis 1 psychiatric disorder (DSM-IV, [43]), including ‘substance abuse’; (3) no clinically significant medical disease; (4) no use of medication; (5) no family history of alcoholism and/or substance use disorder. Cocaine-free polydrug controls met the same criteria except that they reported no history of past or current cocaine use. Participants were selected by means of a phone interview by a research assistant with the M.I.N.I. [44], a brief diagnostic tool that screens for several psychiatric disorders. The sample was obtained from a pool of approximately 60 potential volunteers who responded to the advertisement for studies conducted in our lab over the period of one year. Within this pool of potential volunteers, the most common reason for excluding an individual from the study were hints to a psychiatric disorder (ADHD, mania) and/or medication use.

Participants were asked to refrain from taking all psychoactive drugs for at least two weeks, not to consume alcohol on the night before the experimental session and to have a normal night rest. Participants' compliance with the instruction was encouraged by taking a (not further analyzed) saliva sample at the beginning of the session (cf., [45]).

Participants in the two groups were matched for race (100% Caucasian), age, sex and IQ (measured by Raven's Standard Progressive Matrices; SPM: [46]) and alcohol consumption. Although cocaine was the preferred drug of use for the participants, all 18 were also polydrug users. All cocaine users also reported cannabis use, 16 had used MDMA. All cocaine users reported to have never used LSD, barbiturates, steroids, solvents or opiates, and they consumed alcohol on at least a weekly basis. Demographic and drug use statistics are provided in Tables 1 and 2. Written informed consent was obtained from all participants after the nature of the study was explained to them; the protocol and the remuneration arrangements of 30 Euro were approved by the institutional review board (Leiden University, Institute for Psychological Research).

**Table 2:** Self-reported use of cocaine.

Sample	Mean (SD)
*Highest regular frequency (times per month)*	5.5 (3.7)
*Highest amount in a 12-h period (peak; grams)*	1.21 (0.44)
*Monthly grams*	1.81 (1.67)
*Lifetime exposure grams*	149 (121)
*Monthly money cocaine (Euro)*	90.5 (83.5)

Standard deviation in parentheses.

### Apparatus and Stimuli

Responses were made by pressing the “Z” or “?” of the QWERTY computer keyboard with the left and right index finger, respectively. The target stimuli were adopted from Huizinga, Dolan, and van der Molen [38], and consisted of geometric figures. Larger (global) rectangles/squares consisted of smaller (local) rectangles or squares. Global stimuli (i.e., squares or rectangles; 93×93 pixels or 93×189 pixels respectively) were composed of many smaller “local” stimuli (i.e., squares or rectangles; 21×21 pixels or 8×46 pixels respectively). The space between the local elements of a stimulus was 3 pixels. A global square consisted of 16 small squares or 8 small rectangles; a global rectangle consisted of 32 small squares or 16 small rectangles.

### Procedure and Design

All participants were tested individually and completed the affect grid, the intelligence test and the Global-Local Task.

The affect grid [32] permits participants to express their mood state on a nine-by-nine matrix varying along the dimensions of valence (1 = extremely negative, 9 = extremely positive) and arousal (1 = low arousal, 9 = high arousal).

Individual IQ was determined by means of a 30-min reasoning-based intelligence test (Raven's Standard Progressive Matrices: SPM [46]). Each item of this test consists of a pattern or sequence of a diagrammatic puzzle with one piece missing. The task is to complete the pattern or sequence by choosing the correct missing piece from a list of options. The items are getting more difficult as the test taker proceeds through the test. The SPM assesses the individual's ability to create perceptual relations and to reason by analogy independent of language and formal schooling; it is a standard, widely-used test to measure Spearman's g factor and of fluid intelligence in particular.

In the Global-Local Task (cf., [36]), participants responded to randomly presented rectangles or squares by pressing a left or right response button, respectively. Larger (global) rectangles/squares consist of smaller (local) rectangles or squares. Participants responded to the global shape in one block and to the local shape in another(blocks 1 and 2, in randomized order; 30 practice trials and 100 experimental trials per block). A cue indicated to which dimension (global or local) the participants should respond. Cues that signalled the global (local) dimension consisted of a large (small) square, presented at one side of the target stimulus, and a large (small) rectangle, presented at the other side of the target stimulus. The color of cues and target was red. They remained on the screen until a response was given or 3500 ms had passed. The time interval between presentation of the cue and of the target stimulus was 500 ms. The interval between the response and the presentation of the cue was fixed at 1000 ms. The main dependent variable was the median response latency to local and global targets.

## References

[1] European monitoring centre for drugs and drug addiction (2007). *The state of the drugs problem in Europe, Annual Report 2007.*. http://www.emcdda.europa.eu/.

[2] SAMHSAOffice of Applied StudiesNational Survey on Drug use and Health (2005). http://www.samhsa.gov.

[3] Volkow ND, Fowler JS, Wang GJ (1999). Imaging studies on the role of dopamine in cocaine reinforcement and addiction in humans.. J Psychopharmacol.

[4] Muller CP, Carey RJ, Huston JP, De Souza Silva MA (2007). Serotonin and psychostimulant addiction: focus on 5-HT1A-receptors.. Prog Neurobiol.

[5] Kalivas PW, McFarland K, Bowers S, Szumlinski K, Xi ZX, Baker D (2003). Glutamate transmission and addiction to cocaine.. Ann NY Acad Sci.

[6] Bolla KI, Cadet J, London ED (1998). The neuropsychiatry of chronic cocaine abuse.. J Neuropsych Clin N.

[7] Bolla KI, Ernst M, Kiehl KA, Mouratidis M, Eldreth DA (2004). Prefrontal cortical dysfunction in abstinent cocaine abusers.. J Neuropsych Clin N.

[8] Hester R, Garavan H (2004). Executive dysfunction in cocaine addiction: Evidence for discordant frontal, cingulate, and cerebellar activity.. J Neurosci.

[9] Miller EK (2000). The prefrontal cortex and cognitive control.. Nat Rev Neurosci.

[10] Fillmore MT, Rush CR (2002). Impaired inhibitory control of behaviour in chronic cocaine users.. Drug Alcohol Depend.

[11] Verdejo-Garcia A, Bechara A, Recknor E (2006). Executive dysfunction in substance dependent individuals during drug use and abstinence: An examination of the behavioural, cognitive, and emotional correlates of addiction.. J Int Neuropsych Soc.

[12] Verdejo-Garcia AJ, Perez-Garcia M (2007). Profile of executive deficits in cocaine and heroin polysubstance users: common and differential effects on separate executive components.. Psychopharmacol.

[13] Franken IHA, van Strien JW, Franzek EJ, van de Wetering BJ (2007). Error-processing deficits in patients with cocaine dependence.. Biol Psychol.

[14] Kübler A, Murphy K, Garavan H (2005). Cocaine dependence and attention switching within and between verbal and visuospatial working memory.. Eur J Neurosci.

[15] Colzato LS, van den Wildenberg WPM, Hommel B (2008). Reduced spontaneous eye blink rates in recreational cocaine users: Evidence for dopaminergic hypoactivity.. PLoS ONE.

[16] Kleven MS, Koek W (1996). Differential effects of direct and indirect dopamine agonists on eye blink rate in cynomolgus monkeys.. J Pharmacol Exp Ther.

[17] van der Post J, de Waal PP, de Kam ML, Cohen AF, van Gerven JMA (2004). No evidence of the usefulness of eye blinking as a marker for central dopaminergic activity.. J Psychopharmcol.

[18] Colzato LS, van den Wildenberg WPM, Hommel B (2007). Impaired Inhibitory Control in Recreational Cocaine Users.. PLoS ONE.

[19] Logan GD, Dagenbach D, Carr TH (1994). On the ability to inhibit thought and action: A users' guide to the stop signal paradigm.. Inhibitory processes in attention, memory and language.

[20] Colzato LS, Hommel B (2008). Cannabis, cocaine, and visuomotor integration: Evidence for a role of dopamine D1 receptors in binding perception and action.. Neuropsychologia.

[21] Colzato LS, Hommel B (2009). Recreational use of cocaine eliminates Inhibition of Return.. Neuropsychology.

[22] Posner MI, Cohen Y, Bouma H, Bouwhuis DG (1984). Components of visual orienting.. Attention and performance X: Control of language processes.

[23] Colzato LS, Huizinga M, Hommel B (2009). Recreational use of cocaine impairs cognitive flexibility but not working memory..

[24] Nader MA, Morgan D, Gage HD, Nader SH, Calhoun TL (2006). PET imaging of dopamine D2 receptors during chronic cocaine self-administration in monkeys.. Nat Neurosci.

[25] Bechara A (2005). Decision making, impulse control and neurocognitive perspective.. Nat Neurosci.

[26] Verdejo-Garcia AJ, Lawrence AJ, Clarke L (2008). Impulsivity as a vulnerability marker for substance-use disorders: Review of findings from high-risk research, problem gamblers and genetic association studies.. Neurosci Biobehav R.

[27] Boulougouris V, Tsaltas E (2008). Serotonergic and dopaminergic modulation of attentional processes.. Prog Brain Res.

[28] Navon D (1977). Forest before trees: The precedence of global features in visual perception.. Cognitive Psych.

[29] Yovel I, Revelle W, Mineka S (2005). Who sees trees before forest? The obsessive-compulsive style of visual attention.. Psychol Sci.

[30] Rowe G, Hirsh JB, Anderson AK (2007). Positive affect increases the breadth of attentional selection.. P NATL ACAD SCI USA.

[31] Ashby FG, Valentin VV, Turken AU, Moore S, Oaksford M (2002). The effects of positive affect and arousal on working memory and executive attention: Neurobiology and computational models.. Emotional cognition: From brain to behavior.

[32] Russell JA, Weiss A, Mendelsohn GA (1989). The affect grid: a single- item scale of pleasure and arousal.. J Pers Soc Psychol.

[33] Davis K, Kahn R, Ko G, Davidson M (1991). Dopamine in schizophrenia: A review and reconceptualization.. Am J Psych.

[34] Pooley EC, Fineberg N, Harrison PJ (2007). The met158 allele of catechol-O-methyltransferase (COMT) is associated with obsessive-compulsive disorder in men: case–control study and meta-analysis.. Mol Psyc.

[35] Tripp G, Wickens JR (2007). Dopamine transfer deficit: A neurobiological theory of altered reinforcement mechanisms in ADHD.. J Child Psychol and Psy.

[36] Huizinga M, Dolan CV, van der Molen MW (2006). Age-related change in executive function: Developmental trends and a latent variables analysis.. Neuropsychologia.

[37] Verkes RJ, Gijsman HJ, Pieters MSM, Schoemaker RC, de Visser S (2001). Cognitive performance and serotonergic function in users of ecstasy.. Psychopharmacol.

[38] Verdejo-Garcia AJ, Lopez-Torrecillas F, Aguilar de Arcos F, Perez-Garcia M (2005). Differential effects of MDMA, cocaine, and cannabis use severity on distinctive components of the executive functions in polysubstance users: A multiple regression analysis.. Addict Behav.

[39] Alting von Geusau N, Stalenhoef P, Huizinga M, Snel J, Ridderinkhof KR (2004). Impaired executive function in male MDMA (“ecstacy”) users.. Psychopharmacology.

[40] Ridderinkhof KR, de Vlugt Y, Bramlage A, Spaan M, Elton M, Snel J, Band GPH (2002). Alcohol consumption impairs detection of performance errors in mediofrontal cortex.. Science.

[41] Colzato LS, Fagioli S, Erasmus V, Hommel B (2005). Caffeine, but not nicotine enhances visual feature binding.. EJN.

[42] Logan GD, Gordon RD (2001). Executive control of visual attention in dual-task situations.. Psychol Rev.

[43] American Psychiatric Association (1994). Diagnostic and Statistical Manual of Mental Disorders (4th edition).

[44] Lecrubier Y, Sheehan DV, Weiller E, Amorim P, Bonara I (1997). The Mini International Neuropsychiatric Interview (MINI). A short diagnostic structured interview: reliability and validity according to the CIDI.. Eur Psychiat.

[45] Colzato LS, Erasmus V, Hommel B (2004). Moderate alcohol consumption in humans impairs feature binding in visual perception but not across perception and action.. Neurosci Lett.

[46] Raven JC, Court JH, Raven J (1988). Manual for Raven's progressive matrices and vocabulary scales.

